# Generation and Functional Characteristics of CRISPR/Cas9-Edited *PtrPHOTs* Triple-Gene Mutants in Poplar

**DOI:** 10.3390/plants14101455

**Published:** 2025-05-13

**Authors:** Hongtao Yao, Jiyao Cheng, Yuning Jing, Siran Zhu, Chong Wang, Yuxiang Cheng

**Affiliations:** Key Laboratory of Tree Genetics and Breeding, Northeast Forestry University, Harbin 150040, China; hongtaoyao0202@163.com (H.Y.); chengjiyao1993@163.com (J.C.); j20000505@163.com (Y.J.); imabcab@163.com (S.Z.); nefuwangchong@126.com (C.W.)

**Keywords:** *Populus trichocarpa*, blue light receptors, triple-gene knockouts, Cas9/gRNA

## Abstract

Phototropins (PHOTs), as blue light receptors, play a pivotal role in plant light signal perception and adaptive regulation, yet their functional characteristics in trees remain poorly understood. In this study, the PHOT gene family was identified in *Populus trichocarpa*, and it included three members, *PtrPHOT1*, *PtrPHOT2.1*, and *PtrPHOT2.2*, all of which were highly expressed in mature leaves. Using CRISPR/Cas9 gene editing technology, triple-gene mutations in the *PtrPHOT1/2.1/2.2* (*PtrPHOTs*) were generated, providing initial insights into the functions of PHOTs in trees. Compared to the wild type (WT), triple-gene *ptrphots* mutants displayed curved and wrinkled leaves, reduced leaf area, and delayed phototropic responses, indicating the central role of PHOTs in blue light signal perception. The stomatal aperture recovery rate in mutants was only 40% of that observed in WT, accompanied by significant downregulation of the *BLUS1* gene transcription levels, confirming the conservation of the PHOT-BLUS1-H⁺-ATPase signaling axis in stomatal regulation. Transcriptome of triple-gene *ptrphots* mutants revealed 1413 differentially expressed genes, of which were enriched in auxin response (upregulation of *SAUR* family genes), jasmonic acid (downregulation of *JAZ* genes), and light signaling pathways, suggesting that PHOTs could regulate plant adaptability by integrating light signals and hormone homeostasis. Overall, this study achieved the knockouts of three *PtrPHOTs* family genes, and characteristics of triple-gene *ptrphots* mutants elucidated the multifunctional roles of PHOTs in leaf development, phototropism, and stomatal movement in poplar. Our work provides a foundation for deciphering light signaling networks and molecular breeding in woody plants.

## 1. Introduction

Light is a crucial environmental factor in plant growth and development, as it not only provides energy for photosynthesis but also regulates plant morphogenesis, physiological adaptation, and environmental responses through photoreceptor-mediated signaling networks [1,2]. Plants have evolved multiple classes of photoreceptors to precisely perceive light signals across different wavelengths [1], including red/far-red light receptors (phytochromes, PHYs [3]), blue light receptors (cryptochromes, CRYs [4]; phototropins, PHOTs [5]; ZTL/FKF1/LKP2 family—ZEITLUPE/FLAVIN-BINDING KELCH REPEAT F-BOX 1/LOV KELCH PROTEIN 2 [6]), and ultraviolet light receptors (UV resistance locus 8, UVR8 [7]). Among these, the blue light receptor phototropins (PHOTs) play a critical role in phototropism, stomatal movement, chloroplast positioning, and leaf expansion [5], making them a focal point in light signaling research.

Phototropism is a traditional reaction in which plants orient their growth toward a light source by the bending of stems or leaves. This activity is mainly mediated by the blue light-activated PHOT signaling pathway [8]. PHOTs regulate the polar transport of auxin, inducing differential cell elongation on opposite sides of the organ, ultimately driving phototropic bending [9]. Additionally, PHOTs-mediated stomatal opening [10,11] directly influences plant carbon assimilation and water-use efficiency, while their regulation of chloroplast avoidance movement minimizes photo-oxidative damage and optimizes photosynthetic efficiency [12,13,14]. These multifaceted roles establish PHOTs as central molecular hubs linking light signaling to plant adaptive regulation.

Phototropins are a class of blue light receptor kinases containing light-oxygen-voltage (LOV) domains [15]. They feature two FMN-binding LOV domains (LOV1 and LOV2) at the N-terminus and a serine/threonine kinase (STK) domain at the C-terminus [16]. Blue light induces conformational changes in LOV2, activating STK autophosphorylation and subsequently triggering downstream signaling cascades [17]. In the model plant *Arabidopsis thaliana*, functional differentiation exists between *AtPHOT1* and *AtPHOT2*: *AtPHOT1* regulates hypocotyl phototropic bending under a broad range of blue light intensities, while *AtPHOT2* specifically mediates hypocotyl bending under high-intensity blue light [16,18]. The rice (*Oryza sativa*) genome contains three PHOTs members (*OsPHOT1a*, *OsPHOT1b*, and *OsPHOT2*) [19]. *OsPHOT1a* is highly expressed in coleoptiles, whereas *OsPHOT1b* is predominantly expressed in the leaves of dark-grown rice seedlings [20]. Mutants of *osphot1a/b* exhibit defects in chloroplast movement, reduced photosynthetic pigment content, and growth retardation [19].

Although the functions of PHOTs in herbaceous plants have been extensively characterized, their roles in woody plants remain controversial. As a perennial woody model species, *Populus* may exhibit more complex light-responsive mechanisms due to long-term environmental adaptation. Do *Populus* PHOTs possess unique tissue-specific expression patterns? Are their functions conserved with those in herbaceous plants? These questions require more comprehensive investigations to elucidate. Furthermore, interactions between PHOTs and other signaling pathways, such as auxin and jasmonic acid, are rarely reported in woody plants. Studies in *Arabidopsis* have shown that PHOT1 phosphorylates the BLUS1 kinase to activate plasma membrane H⁺-ATPase, driving stomatal opening [21], while the *SAUR* (small auxin-up RNA) family genes, as early auxin-responsive factors [22], mediate the coupling of light signaling and cell elongation. Auxin and light antagonistically regulate the SAUR-PP2C.D-AHA pathway in the hypocotyl, thereby influencing plant elongation growth [23]. However, whether such mechanisms are conserved in *Populus* remains to be validated.

In this study, *Populus trichocarpa* [24] was used as the experimental material to systematically investigate the functional characteristics of the PHOT gene family through multidisciplinary approaches. CRISPR/Cas9 gene editing technology was employed to generate PHOTs triple-gene knockout mutants (*ptrphots*). Phenotypic differences between the mutants and wild-type plants, including plant height, leaf development, phototropism, and stomatal aperture, were compared. Combined with transcriptome sequencing, differentially expressed genes (DEGs) were screened, and Gene Ontology (GO) and Kyoto Encyclopedia of Genes and Genomes (KEGG) enrichment analyses were conducted to elucidate the regulatory effects of PHOTs on light signaling networks and auxin responses. This study provides a foundation for deciphering light signaling networks and genetic improvement in trees.

## 2. Results

### 2.1. Characterization of PHOT Genes in Populus

**Figure 1:**
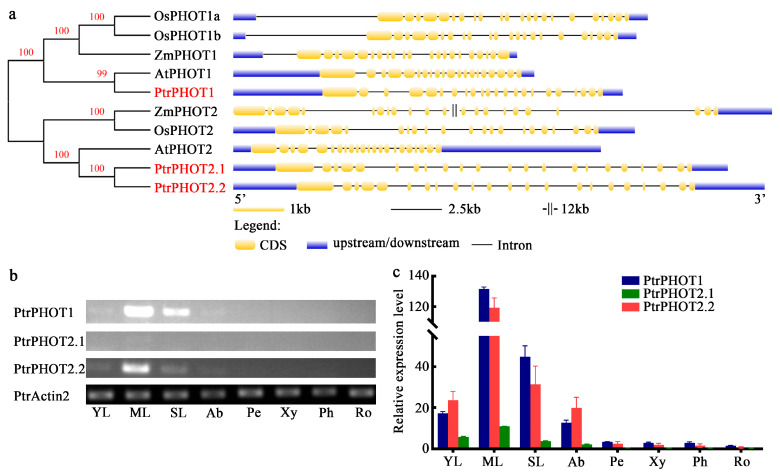
Phylogenetic relationships and gene structure in the PHOT gene family. (**a**) A phylogenetic tree was constructed based on the PHOT family members from *Populus trichocarpa*, *Arabidopsis thaliana*, *Zea mays*, and *Oryza sativa*. The tree was generated using the neighbor-joining (NJ) method in MEGA 11 software with 1000 bootstrap replicates. The gene structures of PHOTs were analyzed using GSDS v2.0, where yellow boxes represent coding sequences (CDS), blue boxes represent non-coding sequences, including upstream and downstream regions, and gray lines indicate introns. (**b**) Expression patterns of *PtrPHOT1*, *PtrPHOT2.1*, *PtrPHOT2.2*, and the reference gene *PtrActin2* [25] in different tissues. YL: young leaves; ML: mature leaves; SL: senescent leaves; Ab: apical buds; Pe: petioles; Xy: xylem; Ph: phloem; Ro: roots. (**c**) Band grayscale values were measured using ImageJ 1.8.0 software and used to calculate relative expression levels. Bar graphs illustrate the relative expression levels of *PtrPHOT1*, *PtrPHOT2.1*, and *PtrPHOT2.2* in different tissues.

In this study, three phototropin genes were identified in *P. trichocarpa* through whole-genome alignment, designated as *PtrPHOT1* (Potri.001G342000), *PtrPHOT2.1* (Potri.009G170600), and *PtrPHOT2.2* (Potri.004G209700). Comparative analysis of the PHOTs gene family in *Arabidopsis*, *Oryza sativa*, and *Zea mays* revealed that these members share similar protein lengths and possess identical numbers of LOV and SKT domains (Table 1). Phylogenetic analysis (Figure 1a) demonstrated that the PHOTs family can be divided into two major evolutionary clades: *PtrPHOT1* clusters with *AtPHOT1* from *Arabidopsis*, *ZmPHOT1* from *Zea mays* and *OsPHOT1a/OsPHOT1b* from *Oryza sativa* (Group I), while *PtrPHOT2.1/2.2* forms a distinct branch with *AtPHOT2*, *ZmPHOT2*, and *OsPHOT2* (Group II). Gene structure analysis indicated that Group I members exhibit a wide range of exon numbers (20–23 exons), whereas Group II members display a more conserved exon count (21–22 exons), suggesting that Group II may be under stronger evolutionary constraint and more functionally conserved.

Tissue-specific expression analysis (Figure 1b,c) revealed that *PtrPHOT1*, *PtrPHOT2.1*, and *PtrPHOT2.2* exhibited the highest expression levels in mature leaves, with relatively high expression in senescent leaves and young leaves, and low expression in apical buds. These genes showed negligible expression in other tissues, including petioles, xylem, phloem, and roots. Notably, the transcription levels of *PtrPHOT1* and *PtrPHOT2.2* are significantly higher than that of *PtrPHOT2.1*, suggesting that *PtrPHOT1* and *PtrPHOT2.2* may play dominant roles in these tissues. The high expression of PHOT genes in mature leaves is likely associated with their active photosynthetic activity. As the primary organs for light capture, mature leaves require PHOTs-mediated blue light perception to regulate chloroplast movement, stomatal opening, and photoprotective mechanisms [5,11,14], thereby optimizing photosynthetic efficiency.

### 2.2. CRISPR/Cas9-Mediated Generation of PtrPHOT1/2.1/2.2 Gene Mutants in P. trichocarpa

**Figure 2:**
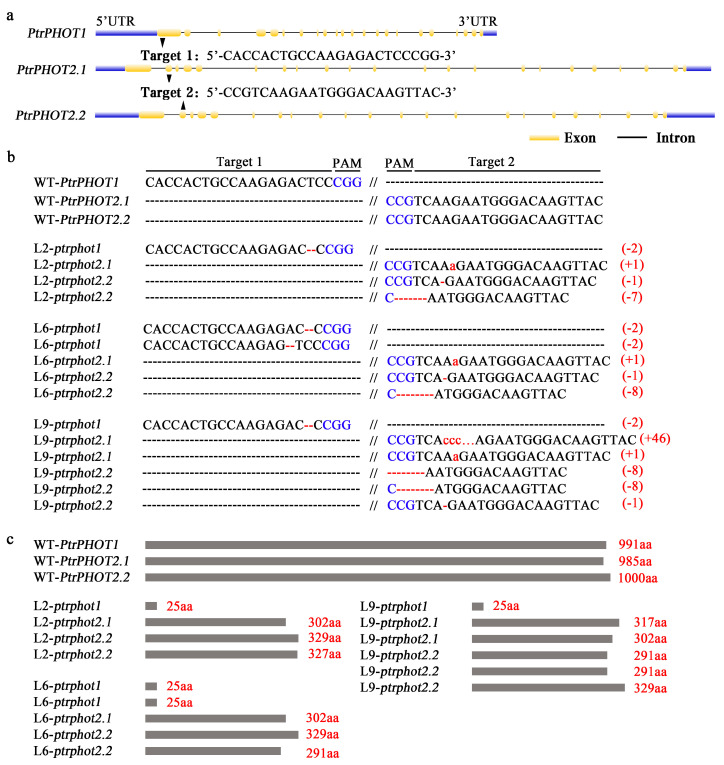
Cas9/gRNA-induced mutations in *PtrPHOT1/2.1/2.2.* (**a**) Design of gRNA target sites for PtrPHOT1/2.1/2.2 genes. (**b**) Sequencing alignment of *PtrPHOT1/2.1/2.2* target sites; blue regions indicate PAM sequences, red “-” represents deletions, and red lowercase letters represent insertions. Numbers in parentheses on the right indicate the number of inserted or deleted bases. (**c**) Deduced amino acid sequences of PtrPHOT1/2.1/2.2 variants with the edited mutations. Editing mutations produced premature stop codons, resulting in drastically reduced amino acid numbers in mutated proteins. Red numbers on the right indicate the number of amino acids.

To elucidate the functional roles of PHOTs gene in *P. trichocarpa*, knockout mutants of the phototropin genes were generated. Given that the PHOTs family has three genes in the *P. trichocarpa* genome, two target sites were designed for *PtrPHOT1* and *PtrPHOT2.1/2.2* to construct the Cas9/gRNA-PtrPHOT1/2.1/2.2 vector. The first target site was located in the first exon of *PtrPHOT1*, and the second target site in the second exon of *PtrPHOT2.1/2.2* (Figure 2a). Cas9/gRNA editing vector of *PtrPHOT1/2.1/2.2* triple genes was successfully constructed (Supplementary Figure S1). After *Agrobacterium*-mediated genetic transformation (Supplementary Figure S2), the hygromycin-resistant positive plants were obtained. Genomic DNA PCR screening using zCas and Hyg-specific primers (for the Cas9 and hygromycin resistance gene, respectively) identified a total of 10 transgenic plants (Supplementary Figure S3).

DNA sequencing of the target sites in 10 transgenic lines revealed that L2, L6, and L9 lines exhibited homozygous/biallelic editing in all three genes (Figure 2b), while the remaining lines were chimeric or partially edited. In line L2, a 2 bp deletion was observed at the *PtrPHOT1* target site, a +1a insertion (frameshift mutation) at the *PtrPHOT2.1* target site, and a compound mutation involving a 1-bp deletion and a 7 bp deletion at the *PtrPHOT2.2* target site. Lines L6 and L9 exhibited similar editing patterns to L2; however, line L9 showed a +46-bp insertion at the *PtrPHOT2.1* target site (Figure 2b). Amino acid sequence deduction indicated that all effective edits resulted in premature termination codons (Figure 2c). The amino acid lengths of the mutated *PtrPHOTs* in these lines were less than one-third of those in the WT, leading to severely disrupted protein sequences and the successful generation of these triple-gene knockout mutants.

### 2.3. Phenotypes of Ptrphots Triple-Gene Mutants

**Figure 3:**
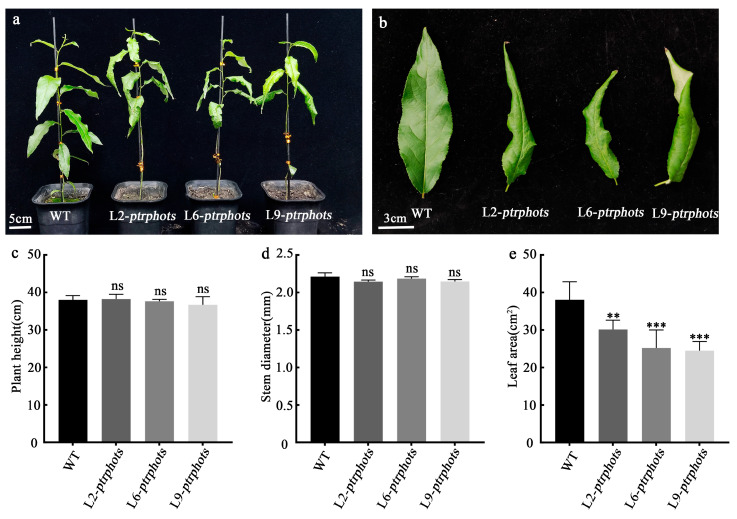
Phenotypic analysis of *ptrphots* mutants (**a**) Phenotypic comparison of 2-month-old wild-type (WT) and mutant (*ptrphots*) plants grown in the greenhouse (scale bar: 5 cm). (**b**) Morphological comparison of the fourth leaf, showing that mutant leaves are curved, narrow, and wrinkled (scale bar: 3 cm). (**c**,**d**) Comparison of plant height and internode diameter between WT and mutants, showing no significant differences. (**e**) Leaf area of mutants was significantly smaller than that of WT. Student’s *t*-test: ^ns^ *p* > 0.05, ** *p* < 0.01, *** *p* < 0.001.

Wild-type and *ptrphots* mutant tissue-cultured plantlets were transplanted into the soils in a greenhouse and observed after two months. Comparative analysis revealed no significant differences in plant height and stem diameter between the mutants and wild-type plants (*p* > 0.05) (Figure 3a,c,d). However, leaf development in the mutants was markedly abnormal. Comparison of the third and fourth leaves showed that the leaf area of the mutants was reduced by approximately 20–35% compared to the WT, with the mutant leaves exhibiting pronounced curling and wrinkling (Figure 3b,e). These results indicate that the knockout of the phototropin receptor genes *PtrPHOT1*, *PtrPHOT2.1*, and *PtrPHOT2.2* disrupts normal leaf development in *P. trichocarpa* but does not affect radial or longitudinal growth of the plants.

### 2.4. Knockout of PtrPHOTs Impairs Phototropic Responses in Populus

**Figure 4:**
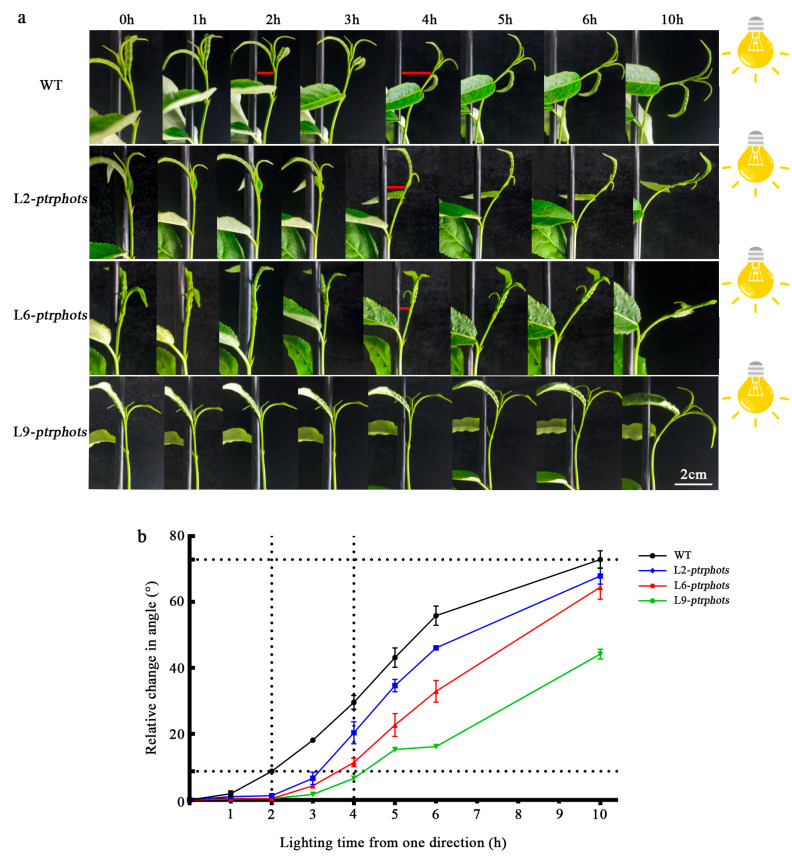
Phototropic response and bending angle dynamics of *ptrphots* mutants (**a**) Schematic diagram of unilateral light treatment (light source on the right side). Red lines indicates a significant change in the bending angle at this point. (**b**) Phototropic bending angles of wild-type (WT) and mutant (*L2-ptrphots*, *L6-ptrphots*, *L9-ptrphots*) plants over time (0–10 h).

Phototropins are known to influence phototropic responses in *Arabidopsis*. To determine the functional conservation of these genes across species, *ptrphots* mutants and WT plants were subjected to unilateral light treatment under dark conditions. Prior to the experiment, all plants exhibited upright apical buds under normal growth conditions. Unilateral light was then applied from the right side. The results demonstrated that, under unilateral light, all plants bent toward the light source; however, the phototropic bending response in *ptrphots* mutants was significantly delayed compared to the WT (Figure 4a). After 2 h of unilateral light exposure, the WT exhibited pronounced bending, while the *ptrphots* mutants showed minimal bending (Figure 4a,b). By 4 h of light exposure, the *ptrphots* mutants began to display relatively noticeable bending. As the duration of unilateral light treatment increased, the bending angles of all plants gradually increased. After 10 h, the bending angles of the *ptrphots* mutants gradually approached those of the WT (Figure 4b). These findings indicate that the loss of PHOTs genes significantly reduces the light sensitivity of *P. trichocarpa*, but phototropic capability is still retained.

### 2.5. Effect of PtrPHOTs Knockout on Light-Induced Stomatal Opening in Populus

**Figure 5:**
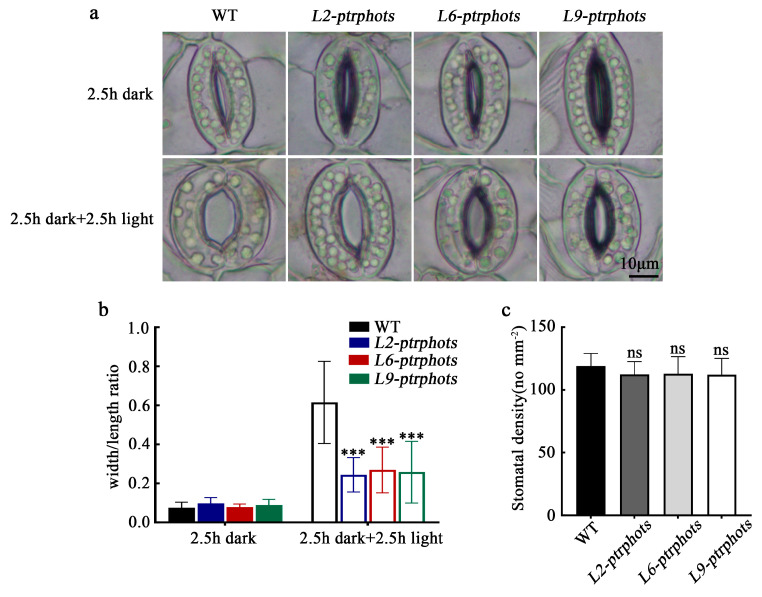
Stomatal aperture and density in WT and *ptrphots* mutants (**a**,**b**) Stomatal phenotypes of wild-type (WT) and mutant (*L2-ptrphots*, *L6-ptrphots*, *L9-ptrphots*) plants after 2.5 h of dark treatment followed by 2.5 h of light treatment under the same conditions. (**c**) Stomatal density statistics of WT and mutants. Scale bar: 10 μm. Student’s *t*-test: ^ns^ *p* > 0.05, *** *p* < 0.001.

To investigate the role of PHOT genes in stomatal regulation in poplar, stomatal aperture and stomatal density were compared between WT and mutants (*L2-ptrphots*, *L6-ptrphots*, *L9-ptrphots*). After 2.5 h of dark treatment, the stomatal aperture (ratio of pore width to pore length) in both WT and mutants decreased to below 0.1, indicating nearly complete closure. Following 2.5 h of light treatment, the stomatal aperture of WT recovered to above 0.6, whereas the aperture of the mutants only recovered to approximately 0.25, demonstrating a significant delay in stomatal reopening in the mutants (Figure 5a,b). Stomatal density analysis revealed that the mutants had slightly lower stomatal density compared to the WT, but the difference was not statistically significant (Figure 5c). These results indicate that PHOTs genes play a critical role in light-induced stomatal opening in poplar.

### 2.6. Transcriptome Analysis Reveals the Impacts of PtrPHOTs Gene Knockout on Leaf Development and Hormonal Homeostasis in Poplar

**Figure 6:**
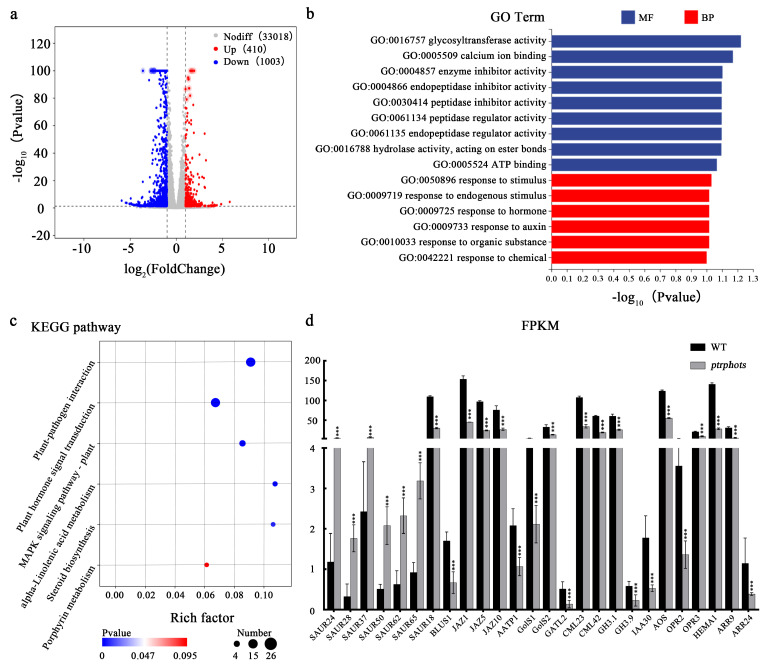
RNA-seq analysis of differentially expressed genes in *ptrphots* mutant leaves (**a**) Volcano plot of differentially expressed genes (DEGs). The x-axis represents log2FoldChange, and the y-axis represents −log_10_ (*p*-value). The two vertical dashed lines indicate the threshold for fold change, and the horizontal dashed line represents the significance threshold. Red dots indicate upregulated genes (Up), blue dots indicate downregulated genes (Down), and gray dots indicate non-significant DEGs (Nodiff). (**b**) GO enrichment analysis. The y-axis represents GO terms, and the x-axis represents −log_10_ (*p*-value) for GO term enrichment. Red bars represent biological processes (BP) and blue bars represent molecular functions (MF). (**c**) KEGG pathway enrichment analysis. The x-axis represents the enrichment factor (rich factor: number of DEGs enriched in the pathway/total number of genes annotated to the pathway), and the y-axis represents KEGG pathways. The size of the dots corresponds to the number of DEGs enriched in the pathway, and the color intensity indicates the significance level. (**d**) Bar graph showing FPKM (Fragments Per Kilobase of transcript per Million mapped reads) [26] expression levels of selected genes in WT and *ptrphots* mutants. The x-axis lists gene names, and the y-axis represents FPKM values. Student’s *t*-test: *** *p* < 0.001.

To systematically elucidate the molecular mechanisms underlying the phenotypic variations in *ptrphots* mutants, this study conducted transcriptome sequencing analysis of mature leaves from WT and three mutant lines (*L2-ptrphots*, *L6-ptrphots*, and *L9-ptrphots*), with three biological replicates per group. Using the DESeq2 method, differentially expressed genes (DEGs) were screened with the criteria of |log2FoldChange| > 1 and FDR-adjusted *p* < 0.05, identifying a total of 1413 DEGs (Table S1). The volcano plot of these DEGs revealed that 410 genes were significantly upregulated, while 1003 genes were significantly downregulated in the mature leaves of *ptrphots* mutants (Figure 6a). To further validate the accuracy of transcriptome data, we randomly selected 14 DEGs from the total DEGs (approximately 1% of the total) for quantitative real-time reverse transcription PCR (qRT-PCR) analysis. The qRT-PCR results showed high consistency with the transcriptome sequencing data in terms of relative expression levels of these genes, strongly confirming the reliability and stability of our transcriptome data (Supplementary Figure S4).

To elucidate the functional roles and associated pathways of these DEGs, GO and KEGG analyses were performed. GO functional enrichment analysis revealed that DEGs were predominantly enriched in two major categories: Biological Process and Molecular Function. Under Biological Process, DEGs were significantly enriched in response to stimulus (GO:0050896), response to hormone (GO:0009725), and response to auxin (GO:0009733) (Figure 6b). In the Molecular Function category, DEGs were notably enriched in pathways related to cell wall polysaccharide biosynthesis, calcium signaling, and energy metabolism, including glycosyltransferase activity (GO:0016757), calcium ion binding (GO:0005509), enzyme inhibitor activity (GO:0004857), and ATP binding (GO:0005524). In mature leaves of *ptrphots* transgenic plants, SAUR-like auxin-responsive protein family genes—associated with auxin responses—were markedly upregulated (SAUR24, SAUR28, SAUR37, SAUR50, SAUR62, and SAUR65), while SAUR18 was downregulated. Members of the Arabidopsis Response Regulator (ARR) family (ARR9, ARR22, and ARR24) were significantly downregulated. Additionally, *BLUS1*, a gene involved in PHOT-mediated blue light signaling, was notably suppressed in the mutants. Genes related to cell wall polysaccharide synthesis, calcium signaling, and energy metabolism (GolS1, GolS2, GATL2, CML23, CML42, and AATP1) were significantly downregulated (Figure 6d).

KEGG pathway analysis further revealed that the DEGs were significantly enriched in plant hormone signal transduction (ko04075), MAPK signaling pathway (ko04016), and porphyrin metabolism (ko00860) (Figure 6c). In mature leaves of *ptrphots* transgenic plants, in addition to the SAUR-like auxin-responsive protein family, key auxin-related genes from the GH3 (GH3.1, GH3.9) and IAA (IAA30) families were significantly downregulated, suggesting disrupted spatial distribution of auxin signaling in the mutants. Core repressors of jasmonate signaling (JAZ1, JAZ5, JAZ10) were also markedly suppressed, potentially leading to enhanced jasmonate responses. Concurrently, key enzymes involved in jasmonate biosynthesis (AOS, OPR2, OPR3) and chlorophyll biosynthesis (HEMA1, CHLG) exhibited significant downregulation (Figure 6d). Both GO and KEGG analyses demonstrated that PHOTs knockout substantially impaired light signal perception, hormone homeostasis, and metabolic regulation networks in *P. trichocarpa* leaves.

## 3. Discussion

### 3.1. PtrPHOTs Genes Knockout Causes Abnormal Leaf Development and Delayed Phototropic Response

This study provides the simultaneous knockout of all PHOT family genes (*PtrPHOT1/2.1/2.2*) in the woody plant *P. trichocarpa*. The mutants exhibited significant leaf wrinkling and reduced leaf area (20–35% reduction), while plant height and stem diameter remained unaffected (Figure 3). These findings suggest that *PtrPHOTs* genes primarily regulate leaf development without significantly influencing stem growth. In contrast to observations in *Arabidopsis*, where *phot1phot2* double mutants display adaxially curled leaves that hang nearly perpendicular to the petiole [16], the *P. trichocarpa* mutants showed adaxial bending accompanied by wrinkling. This phenotypic divergence may reflect evolutionary differences in light signaling networks between woody and herbaceous plants.

In *Arabidopsis*, PHOT1 and PHOT2 redundantly regulate leaf expansion and positioning. Under low-light conditions, PHOT1 alone mediates these processes [27], whereas under high-light conditions, both PHOT1 and PHOT2 function cooperatively [16]. Additionally, downstream factors such as NPH3, PKS2, and RPT2 have been shown to participate in PHOT1/PHOT2-mediated leaf expansion and positioning [28,29]. The reduced leaf area in *P. trichocarpa* mutants aligns with observations in *Arabidopsis* double mutants [30]. However, the leaf wrinkling phenotype in *P. trichocarpa* may involve additional mechanisms beyond those reported in *Arabidopsis* (e.g., NPH3 and PKS2). Transcriptome data revealed significant downregulation of cell wall polysaccharide biosynthesis-related genes (e.g., GolS1, GolS2, GATL2) in *ptrphots* mutants (Figure 6d), suggesting that cell wall impairment might contribute to abnormal leaf expansion and wrinkling. Notably, plant height and stem diameter remained unaffected in *ptrphots* mutants (Figure 3a,c,d), contrasting sharply with *Arabidopsis phot1phot2* mutants, which exhibit a 67% reduction in fresh weight compared to wild-type [29]. The discrepancy may result from divergent light signaling and growth regulation mechanisms between woody and herbaceous plants. In *Arabidopsis*, leaf fresh weight constitutes a major portion of total biomass, and impaired leaf development significantly impacts overall plant growth. In contrast, stem growth in woody plants likely depends more on vascular tissue development rather than light signaling-mediated leaf expansion.

Moreover, *ptrphots* mutants exhibited delayed phototropic responses under unilateral white light but retained partial phototropic capability (Figure 4a,b). This contrasts with *Arabidopsis phot1phot2* double mutants, which completely lose phototropism under blue light [5,14,16]. The phenotypic discrepancy may stem from differences in light quality. While this study employed white light (containing both blue and red wavelengths), most *Arabidopsis* experiments use monochromatic blue light. Apparently, red light can trigger phototropism via phytochromes (PHYs) [31], and cryptochromes (CRYs) have also been implicated in *Arabidopsis* phototropic responses [32]. Thus, red light components in white light and CRY-mediated signaling may partially compensate for the loss of poplar *PtrPHOT* function in phototropism. Future studies employing monochromatic light assays could further dissect the wavelength-specific contributions to phototropism in poplar.

### 3.2. Stomatal Aperture Defects in the Ptrphots Mutants and the Conservation of the PHOT-BLUS1-H⁺-ATPase Signaling Axis

This study investigated the role of PHOTs genes in stomatal regulation in poplar. The *ptrphots* mutants exhibited significantly delayed stomatal aperture recovery following light treatment, while stomatal density remained unchanged (Figure 5). These findings indicate that *PtrPHOTs* genes primarily regulate stomatal movement rather than stomatal development in poplar. Although stomatal aperture recovery was markedly delayed, partial functionality was retained, which might be attributed to the spectral complexity of white light. Since white light contains blue and red wavelengths, and red light can also induce stomatal opening [33,34], the red light component may partially compensate for the loss of *PtrPHOTs*-mediated stomatal regulation in poplar.

*BLUS1* gene expression was significantly downregulated in *ptrphots* mutants (Figure 6d), and its transcript levels positively correlated with stomatal aperture. It implies the evolutionary conservation of the PHOT-BLUS1-H^+^-ATPase signaling axis in plants. In *Arabidopsis*, studies have shown that blue light-activated PHOT1 directly phosphorylates the BLUS1 kinase, and phosphorylated BLUS1 indirectly activates plasma membrane AHA1-type H^+^-ATPase, thereby driving stomatal opening [35]. The phosphorylation status of *BLUS1* is closely linked to stomatal aperture, and the downregulation of *BLUS1* in *ptrphots* mutants likely accounts for the delayed stomatal recovery. Notably, stomata in the mutants did not completely lose responsiveness, retaining partial opening capacity. It suggests that other photoreceptors (e.g., cryptochromes or phytochromes) may partially compensate for the loss of PHOTs function. For instance, in *Arabidopsis*, CRY1 modulates stomatal aperture indirectly via the COP1-SPA complex [36], while phyB participates in red light-induced stomatal opening [37]. Future studies employ monochromatic light assays to dissect the wavelength-specific effects on stomatal regulation in poplar and elucidate the precise roles of cryptochromes and phytochromes in this process.

### 3.3. Transcriptome Analysis Reveals Potential Mechanisms Underlying Leaf Developmental Changes Induced by PtrPHOTs Knockout

Regarding the striking phenotypes of leaf wrinkling and area reduction induced by *PtrPHOTs* knockout, our transcriptome sequencing results suggest the involvement of multifaceted regulatory mechanisms. From the perspective of phytohormone regulation, auxin plays a pivotal role in leaf development and photomorphogenesis, exhibiting complex interactions with blue light signaling. Transcriptomic analysis in this study revealed significant upregulation of SAUR-like auxin-responsive genes in *ptrphots* mutants (Figure 6d). This observation aligns with the pattern observed in *Arabidopsis*, where *SAUR* genes are generally upregulated during enhanced auxin signaling [38]. As the largest family of early auxin-responsive genes in plants, *SAUR* genes have been reported to participate extensively in processes such as cell elongation, organ development, and photomorphogenesis [22,39]. In *Arabidopsis*, moderate overexpression of *SAUR* genes promotes hypocotyl elongation, while excessive expression inhibits elongation [23], demonstrating the complex regulatory role of *SAUR* genes in auxin signaling. In this study, the loss of *PtrPHOTs* function may have disrupted the balance between light signaling and auxin homeostasis, leading to abnormal upregulation of *SAUR* gene expression. This dysregulated auxin signaling could negatively impact normal cell expansion and leaf area formation.

Furthermore, the jasmonic acid (JA) signaling pathway plays a crucial role in plant growth and environmental responses. Our study found that the expression of core repressors in the JA signaling pathway (such as *JAZ1*, *JAZ5*, and *JAZ10*) was significantly downregulated in the mutants (Figure 6d). JAZ proteins typically suppress JA signaling by interacting with transcription factors (e.g., *MYC2*) and corepressors [40]. The downregulation of these genes may lead to derepression of JA signaling, thereby enhancing jasmonate responses. Given the important roles of JA signaling in plant defense and stomatal regulation [41], alterations in this signaling pathway may indirectly affect leaf growth and development.

From the perspective of cell wall synthesis and function, the cell wall plays a critical role in maintaining plant cell morphology and mechanical stability [42]. Transcriptome analysis revealed significant downregulation of genes related to cell wall polysaccharide synthesis (including *GolS1*, *GolS2*, and *GATL2*) in *ptrphots* mutants (Figure 6d). As essential components of the cell wall, polysaccharides are crucial for proper cell wall assembly and functional maintenance. The downregulation of these genes may lead to insufficient polysaccharide synthesis, thereby compromising cell wall integrity, elasticity, and mechanical strength. This could not only restrict normal cell expansion but also make cells more susceptible to mechanical damage during growth, ultimately resulting in leaf curling, wrinkling phenotypes, and limiting leaf area expansion.

From the perspectives of energy metabolism and chloroplast function, the expression of energy metabolism-related genes (e.g., *AATP1*) was significantly downregulated in the mutants. *AATP1*, a plastidial ADP/ATP transporter localized to the chloroplast inner envelope membrane, provides energy support for various metabolic processes within chloroplasts [43]. Its downregulation may impair normal chloroplast functions, including chloroplast photorelocation movement and photosynthetic efficiency. The compromised chloroplast function could negatively affect photosynthetic processes, thereby reducing the accumulation of photosynthetic products, which may consequently restrict leaf growth and area expansion to some extent.

In addition, this study has limitations in not thoroughly analyzing the specific effects of different light qualities on poplar light signaling networks, the interaction mechanisms between *PtrPHOTs* and other photoreceptors, and the direct impact of PHOTs deficiency on chloroplast movement. Future research could employ monochromatic light experiments to investigate the specific effects of different light wavelengths (e.g., red and blue light) on poplar light signaling networks, particularly their roles in stomatal opening and phototropism. By combining chloroplast imaging and other experiments, we could analyze how *PtrPHOTs* knockout affects chloroplast movement and photosynthetic efficiency, exploring its role in light signal regulation in poplar. Moreover, functional divergence among *PHOT* homologs has been observed in *Arabidopsis* and *Oryza sativa*. Phylogenetic and gene structure analyses revealed that *PtrPHOT1* in *Populus* clusters with Group I members (*AtPHOT1*, *OsPHOT1a/b*), while *PtrPHOT2.1/2.2* belong to Group II (*AtPHOT2*, *OsPHOT2*) (Figure 1a, Table 1). These findings suggest potential functional conservation of *PHOTs* in *Populus*. However, this study primarily focused on triple-gene mutants (*ptrphots*), aiming to comprehensively evaluate the overall role of *PHOTs* in light signaling pathways in *Populus*, which simultaneously limited our direct assessment of individual gene functions. In *Arabidopsis*, *AtPHOT1* and *AtPHOT2* exhibit distinct light intensity dependencies in leaf development, phototropism, and chloroplast relocation [16,18,27]. Similarly, analogous functional partitioning may exist in *Populus*. Therefore, in future studies, we plan to generate *PtrPHOT1* single mutants, as well as single and double mutants of *PtrPHOT2.1* and *PtrPHOT2.2*, to elucidate the specific roles of each gene in light signaling transduction in *Populus*.

In conclusion, this study creates the *PHOTs* (*PtrPHOT1/2.1/2.2*) triple-gene knockouts in poplar using CRISPR/Cas9 gene editing. The *ptrphots* mutants exhibit significant phenotypic defects: reduction in leaf area with curved and wrinkled morphology and delayed phototropic responses. Additionally, the light-induced stomatal aperture recovery capability is extremely impaired in mutants, and combined with the significant downregulation of the *BLUS1* gene, suggesting the conservation of the PHOT-BLUS1-H^+^-ATPase signaling axis in poplar. Transcriptome data reveals numerous DEGs enriched in auxin, JA, and light signaling pathways, demonstrating that *PtrPHOTs* regulate plant adaptability by integrating light signals with hormonal homeostasis. Our findings deepen the understanding of light signaling networks in woody plants, which provides the foundations for breeding new forest varieties with high photosynthetic efficiency and stress resistance in trees.

## 4. Materials and Methods

### 4.1. Plant Materials and Growth Conditions

In this experiment, the *Populus trichocarpa* Nisqually-1 genotype was selected as the material for genetic transformation. Both wild-type and transgenic plants were propagated using in vitro tissue culture methods: stem segments were inoculated onto WPM basal medium (PhytoTech Lab, L449, Lenexa, KS, USA) supplemented with vitamins and 2.5% (*w*/*v*) sucrose and adjusted to pH 5.8. The tissue culture conditions included a light intensity of 90 μmol·m^−2^·s^−1^, a temperature of 23–25 °C, and a photoperiod of 16 h light/8 h darkness. Soil-grown seedlings were cultivated in a greenhouse (light intensity of 250 μmol·m^−2^·s^−1^, temperature of 25–28 °C). Thirty-day-old sterile tissue-cultured plantlets were used for *Agrobacterium*-mediated transformation experiments. Three asexual propagation methods—apical bud cloning, lateral bud cloning, and shoot regeneration—were employed for transgenic plants. Apical and lateral buds were excised from young trees, hydroponically cultured until rooting for three weeks, and shoot regeneration propagation was performed as previously described [44]. Two-month-old soil-grown *P. trichocarpa* young trees were used for genetic analysis.

### 4.2. Identification of the PHOT Genes in P. trichocarpa

Based on the protein sequences of PHOTs family members from *Arabidopsis thaliana*, *Zea mays*, and *Oryza sativa* (Table 1), homologous alignment was performed on the *Populus trichocarpa* genome using the Phytozome v13 database [45]. The SMART tool (http://smart.embl-heidelberg.de; accessed on 24 September 2024) [46] was employed to screen candidate genes containing LOV and STK domains. The MEGA11 software was used to construct a phylogenetic tree using the Neighbor-Joining method, with 1000 Bootstrap replicates [47]. Gene structure diagrams were generated using the Gene Structure Display Server 2.0.

### 4.3. gRNA Design and Vector Construction

For the mutations in *PtrPHOT1/2.1/2.2*, we employed the Cas9/gRNA gene editing technology. Two gRNA target sites were manually selected to cover these three genes (primers listed in Supplementary Table S2), and a BLASTN search was conducted against the *P. trichocarpa* genome for each target site to ensure specificity. Candidate target sites were chosen near the 5’-end of the coding sequences (CDS). For the construction of the Cas9/gRNA plant editing vector, we followed the method previously described by Xing [48]. The gRNA fragment was amplified using pCBC-DT1T2 as the template, and the purified DNA fragment was assembled into the pHSE401 plant expression vector, provided by Professor Qi-Jun Chen, using the Golden Gate method. Primers used in the experiment are listed in Supplementary Table S2.

### 4.4. Generation of Transgenic P. trichocarpa

The constructed pHSE401-2gRNA vector was introduced into the *Agrobacterium tumefaciens* strain GV3101 for *P. trichocarpa* genetic transformation experiments. Following the *Agrobacterium*-mediated transformation method described by Li and Xu et al. [25,44], 30-day-old healthy tissue-cultured plantlets of *P. trichocarpa* were used as the genetic transformation material, with hygromycin (Hyg) as the selection marker. The *Agrobacterium* carrying the pHSE401-2gRNA vector was incubated until OD600 = 0.6, and the bacterial suspension was collected. After centrifugation at 2200× *g* for 10 min at 4 °C, the pellet was resuspended in 50 mL of suspension buffer (containing 0.04 g WPM, 1.25 g sucrose, 0.0136 g MES, pH = 5.2). Stem segments of *P. trichocarpa* plantlets, approximately 1.0 cm in length, were gently shaken in the suspension for 20 min for infection. The infected explants were co-cultured for 48 h, then transferred to selection medium containing 10 mg L^−1^ hygromycin under light conditions. After 25 days, the stem explants were transferred to selection medium with 5 mg L^−1^ hygromycin for further cultivation. Hygromycin-resistant shoots were induced after 15 days, excised, and rooted on medium containing 5 mg L^−1^ hygromycin.

### 4.5. Identification of Mutations

To detect Cas9/gRNA-induced mutations, genomic DNA was extracted from the leaves of wild-type and transgenic plants using a Plant Genomic DNA Extraction Kit (Bioteke, Wuxi, China). Genomic DNA PCR was performed using zCas (Cas9 gene-specific primers) and Hyg (hygromycin resistance gene primers) listed in Supplementary Table S2 to confirm transgenic plants. Positive plants were further analyzed by amplifying genomic DNA fragments flanking the gRNA target sites. The PCR products were purified by 1% agarose gel electrophoresis and cloned into the pMD18-T vector (Takara, Dalian, China). For each line, 20 single-colony clones were randomly selected for sequencing (Genesoul, Harbin, China) to identify the edited mutations.

### 4.6. Analyses of Growth and Leaf Area

Wild-type and mutant plants were transplanted into greenhouse soil (light intensity of 250 μmol·m^−2^·s^−1^, temperature of 25–28 °C). After 60 days of growth, plant height (from the pot rim to the apical bud) was measured using a tape measure, and the diameters of the 3rd, 5th, 7th, 9th, 11th, 13th, and 15th internodes were measured using a vernier caliper (mean values from three plant lines were recorded). For leaf area measurement, the 3rd and 4th fully expanded leaves were selected, photographed, and analyzed using ImageJ 1.8.0 software to calculate leaf area.

### 4.7. Extraction of RNA and RT-PCR and qRT-PCR

Total RNA was isolated from samples using pBIOZOL Plant Total RNA Extraction Reagent (Bio-Flux, Hangzhou, China), and RNA purity was assessed (OD260/280 > 1.9, OD260/230 > 1.9). Reverse transcription was performed using the PrimeScript RT Reagent Kit with gDNA Eraser (TaKaRa, Dalian, China) to synthesize cDNA. RT-PCR analysis was conducted using cDNA as the template and primers (Supplementary Table S1) to examine the expression patterns of *PtrPHOT1*, *PtrPHOT2.1*, and *PtrPHOT2.2* in different tissues. PCR products were separated and detected by 1% agarose gel electrophoresis. Gel images were analyzed using ImageJ 1.8.0 software to measure grayscale values, and the relative expression levels of each gene were calculated by comparison with a known concentration marker. qRT-PCR experiments were conducted using a qTOWER3/G system (Analytik Jena, Jena, Thuringia, Germany) with SYBR Green (GenStar, Harbin, China). Each 20 µL reaction mixture contained 10 µL of 2 × RealStar Fast SYBR qPCR Mix, 1 µL of cDNA template, 0.4 µL of ROX Reference Dye, 0.5 µL of each gene-specific primer, and 7.6 µL of distilled H_2_O. PtrActin2 was used as an internal control, and gene expression levels were calculated using the comparative cycle threshold (Ct, 2^−∆Ct^) method. Three biological experiment replicates and three technical experiment replicates were performed for each outcome. All primers used for qRT-PCR are listed in Table S2.

### 4.8. Stomatal Aperture Measurements

The fourth mature leaf from 3-month-old plants was selected, and the abaxial epidermis of the detached leaf was placed in a solution containing 10 mM MES-KOH (pH 6.5) and 30 mM KCl [49]. The samples were kept in the dark at 20 °C for 2.5 h to induce stomatal closure, then transferred to the same solution and exposed to light (250 μmol·m^−2^·s^−1^) for 2.5 h. The abaxial epidermis was peeled off, and stomatal images were captured under a confocal microscope. Stomatal aperture and pore length were measured using ImageJ 1.8.0, and the aperture-to-pore length ratio was calculated as an indicator of stomatal opening. Fifty stomata were measured for each group.

### 4.9. Transcriptome Sequencing

For RNA sequencing of leaves, the fourth leaf with reduced leaf area and wrinkling phenotypes from 4-month-old WT and *ptrphots* transgenic lines (*L2-ptrphots*, *L6-ptrphots*, *L9-ptrphots*) was collected, with three biological replicates for each genotype. RNA sequencing analysis was performed at Personalbio (Shanghai, China). Raw reads containing adapters, poly-N sequences, or low-quality reads were removed, and the remaining reads were aligned to the *P. trichocarpa* v3.0 genome. Gene expression levels were calculated using the FPKM (Fragments Per Kilobase of transcript per Million mapped reads) method [26], and differential gene expression patterns were detected using the DESeq2 method. Gene Ontology (GO) and Kyoto Encyclopedia of Genes and Genomes (KEGG) analyses of the DEGs were conducted on the website (https://www.genescloud.cn/home; accessed on 18 January 2025).

### 4.10. Statistical Analysis

All data analyses and statistical tests were conducted by SPSS version 24.0. Values are displayed as the means ± standard deviation (SD), and the number of asterisks indicates statistical significance at different levels (* *p* < 0.05, ** *p* < 0.01 and *** *p* < 0.001).

## Figures and Tables

**Table 1:** PHOTs gene family in *Arabidopsis*, *P. trichocarpa*, *Zea mays*, and *Oryza sativa*.

Name	Gene ID	Protein Length (aa)	LOV Number	STK Number
*AtPHOT1*	AT3G45780	996	2	1
*AtPHOT2*	AT5G58140	915	2	1
*PtrPHOT1*	Potri.001G342000	991	2	1
*PtrPHOT2.1*	Potri.009G170600	985	2	1
*PtrPHOT2.2*	Potri.004G209700	1000	2	1
*ZmPHOT1*	Zm00001d044599	911	2	1
*ZmPHOT2*	Zm00001d032353	932	2	1
*OsPHOT1a*	LOC_Os11g01140	921	2	1
*OsPHOT1b*	LOC_Os12g01140	921	2	1
*OsPHOT2*	LOC_Os04g23890	907	2	1

## Data Availability

Data are contained within the article and Supplementary Materials.

## Supplementary Materials

The following supporting information can be downloaded at: https://www.mdpi.com/article/10.3390/plants14101455/s1, Figure S1: Construction of a Cas9/gRNA-PtrPHOT1/2.1/2.2 triple genes editing vector; Figure S2: Transformation of *P. trichocarpa* with *pHSE401-PtrPHOT* (gRNA); Figure S3: Molecular identification of 10 transgenic and wild-type plants was carried out with zCas and Hyg primers; Figure S4: qRT-PCR validation of RNA-seq data reliability. Table S1: List of differentially expressed genes in the *ptrphots* mutants compared with wild-type plants. Table S2: Primer sequences used in this study.
