# Supplementary material for: Generation and Functional Characteristics of CRISPR/Cas9-Edited PtrPHOTs Triple-Gene Mutants in Poplar
Source: Plants (Basel). 2025 May 13;14(10):1455. doi: 10.3390/plants14101455 (PMC12114965; doi:10.3390/plants14101455)
Supplement: Supplementary file 1 [file plants-14-01455-s001.zip › Supplementary Figure1-4.pdf]

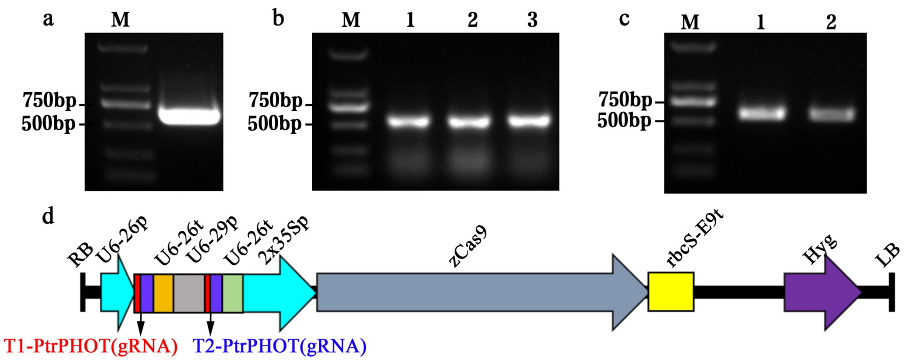

Figure S1. Construction of a Cas9/gRNA-PtrPHOT1/2.1/2.2 triple genes editing vector. (**a**) PCR amplification of the fragment containing dual gRNAs; M represents the marker (DL2000). (**b**) Identification of positive colonies (1, 2, and 3) by PCR after transforming the ligation product into E. coli. (**c**) PCR verification of Agrobacterium GV3101 colonies 1 and 2, with the target band around 600 bp. (**d**) Structure of the T-DNA region in the pHSE401 vector, containing the dual gRNA expression cassette and the hygromycin resistance gene (Hyg).

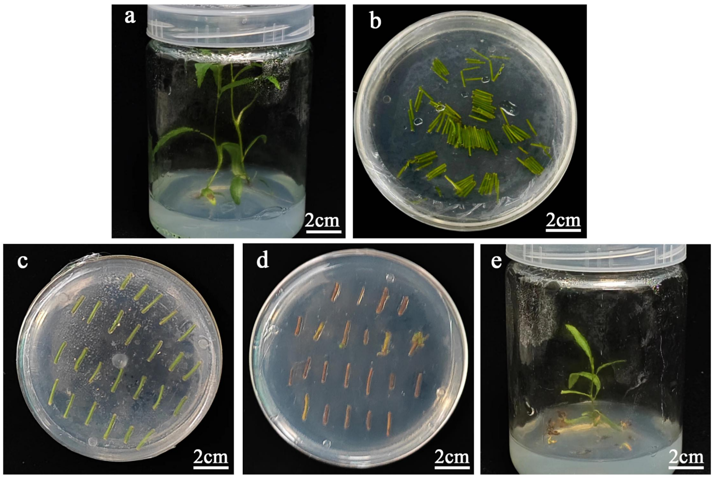

Figure S2. Transformation of *P.trichocarpa* with *pHSE401-PtrPHOT*（gRNA）. (**a**)Wild-type *Populus trichocarpa* aseptic tissue-cultured seedlings at 30 days of growth. (**b**) Stem segments co-cultured with *Agrobacterium* for 48 hours. (**c**) Culture in differentiation selection medium containing 10 mg/L Hyg for approximately 25 days. (**d**) Culture in differentiation selection medium with 5 mg/L Hyg for about 15 days, showing partial bud browning and some buds in good condition. (**e**) Morphology after approximately one month of culture in rooting medium containing 5 mg/L Hyg. Scale bar: 2 cm.

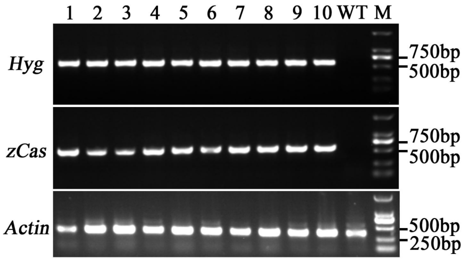

Figure S3. Molecular identification of 10 transgenic and wild-type plants was carried out with zCas and Hyg primers. Actin reference gene amplification: The target bands were successfully amplified in both the wild-type (WT) and 10 positive lines (1-10), indicating successful genomic DNA extraction. Hyg resistance gene and zCas gene detection: No amplification was observed in WT, while all positive lines (1-10) showed amplification of the target bands, confirming that these 10 positive lines are transgenic plants. M represents the marker (DL2000).

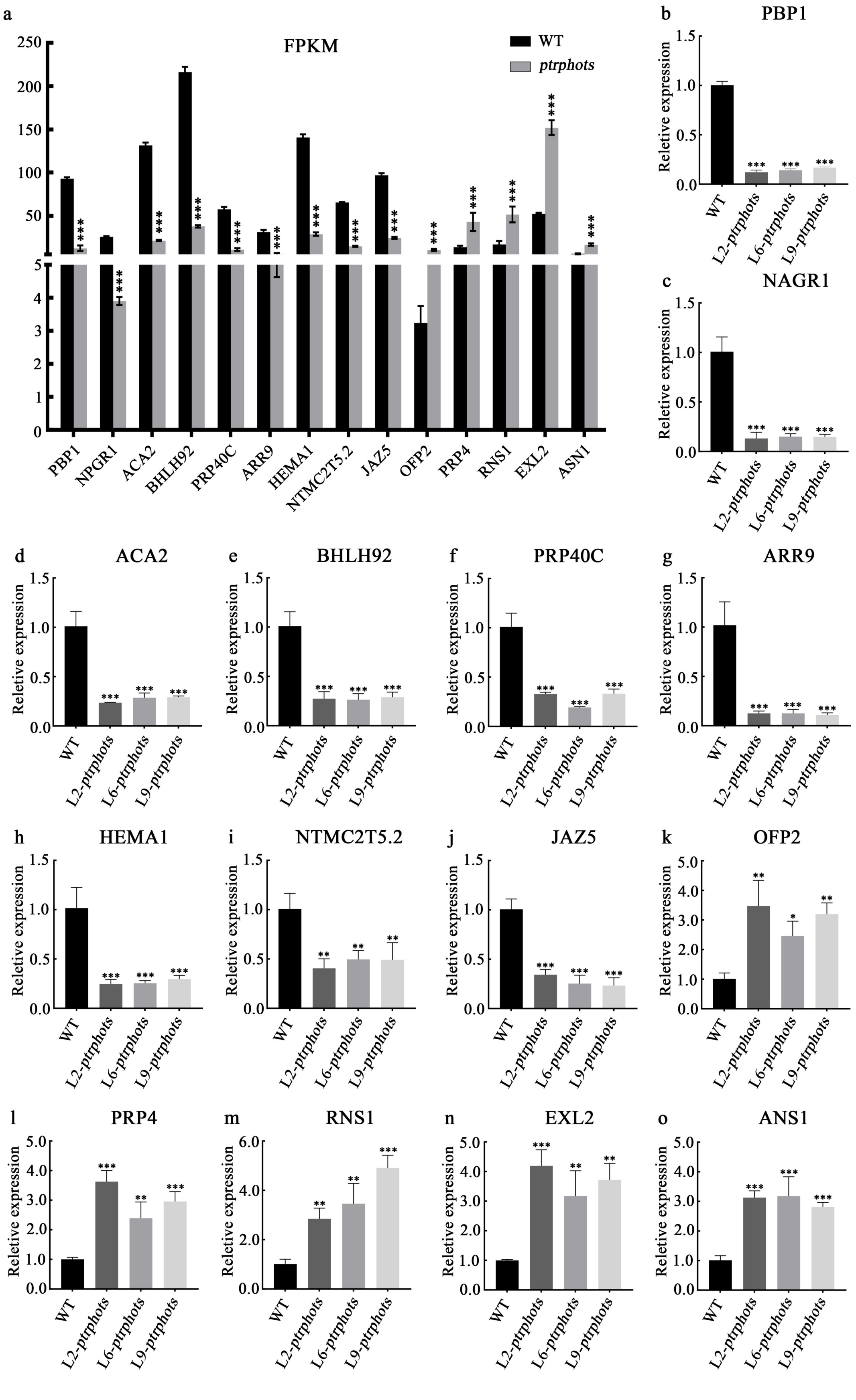

Figure S4. qRT-PCR validation of RNA-seq data reliability. (a) The bar graph displays FPKM expression levels of 14 randomly selected genes from the 1,413 differentially expressed genes (DEGs) identified by

transcriptome analysis in wild-type (WT) and *ptrphots* mutants. The x-axis indicates gene names, while the y-axis represents FPKM values. (b-o) Quantitative real-time reverse transcription PCR (qRT-PCR) analysis of relative expression levels for 14 DEGs (PBP1, NAGR1, ACA2, BHLH92, PRP40C, ARR9, HEMA1, NTMC2T5.2, JAZ5, OFP2, PRP4, RNS1, EXL2, ASN1) in WT and three mutant lines (*L2-ptrphots*, *L6-ptrphots*, *L9-ptrphots*). All experiments were performed with three biological replicates. Data are presented as mean ± SD ($n = 3$). Statistical significance was determined by Student's t-test: *$p < 0.05$, **$p < 0.01$, ***$p < 0.001$.
