# Supplementary material for: Generation and Functional Characteristics of CRISPR/Cas9-Edited PtrPHOTs Triple-Gene Mutants in Poplar
Source: Plants (Basel). 2025 May 13;14(10):1455. doi: 10.3390/plants14101455 (PMC12114965; doi:10.3390/plants14101455)
Supplement: Supplementary file 1 [file plants-14-01455-s001.zip › Table S2.pdf]

Table S2. Primer sequences used in this study

Primer name	Primer use	Primer sequence(5′-3′)
DT1-BsF	Target site gRNA	ATATATGGTCTCGATTGACCACTGCCAAGAGACTCCGTT
DT1-F0		TGACCACTGCCAAGAGACTCCGTTTTAGAGCTAGAAATAGC
DT2-R0		AACTCAAGAATGGGACAAGTTACAATCTCTTAGTCGACTCTAC
DT2-BsR		ATTATTGGTCTCGAAACTCAAGAATGGGACAAGTTACAA
PHOT 1-F/R	Identifying editing site	TTGAGTAGTAGCGGTAGCGGAG；GCAGTGGGGGATTGAGAGACAT
PHOT 2.1-F/R		ATTACTTTTGCGTTGCTAGAGATA；AAGATTGGATAGTCTGGTTTTGTG
PHOT 2.2-F/R		CTCGTCCCGTCGTGTGTGTATG；AAGCCTTAGCCTTCTGACGAGC
zCas-F/R	Transgenic identification	TGAGAACATCGTCATTGAGATGG；TCAGCTTGTCATTCTCATCGTAC
Hyg-F/R		ATGAAAAAGCCTGAACTCACCGC；GCAATCGCGCATATGAAATCACG
PtrActin2-F/R	Reference gene Actin	AACATGGGATTGTTAGCAACTGG；TCCATCACCAGAATCCAGCACA
RT-PHOT 1-F/R	Semi-quantitative RT-PCR primers	GAAGTATTCAACCCCTCCAGT；CAGTAAGTACCCTCTCCTCGT
RT-PHOT 2.1-F/R		GGTTCTATGCTGCGGAGGTTG；CTAACAGGGATGCTGCTTGGG
RT-PHOT 2.2-F/R		CACCGCACCTGGCAGACTCAC；TCACCCTTTTGGTCACGCATA
qRT-PBP1-F/R	qRT-PCR primers	GAAGGGTTGATAAACGAGC；CCCCATCACGGTCAAGATC
qRT-NPGR1-F/R		GCTGCAAAGGAATGCAGAAT；ATTCCATGGCCTGACAAGAG
qRT-ACA2-F/R		GTCTCCCCAAGCGACTATAA；ATTCAGTAGATCACTATCCG
qRT-BHLH92-F/R		TTCTTTCAAGACGAGTGGCAA；ACCAGGCTCTTGAGCTCCAA
qRT-PRP40C-F/R		TAATTGCTGAAGCAGCAGCC；CTTCTGATCCGGTGAAGACT
qRT-ARR9-F/R		TGCTGTTGACGACTGCCTTAT；TTCTCCATTCAAGCCCAAA
qRT-HEMA1-F/R		TATCATTACTGGGAAACGG；CTTTAGCAGCCAAGTGTTT
qRT-OFP2-F/R		GCTCTTGCTACCGTCTCAT；TAGCAGGCTTTGTTACGATTG
qRT-PRP4-F/R		CCGCTTCCATTGCCTACAT；GGAAGACCTTTGGTGGGAG
qRT-NTMC2T5.2-F/R		ACACCCGATACCTTCACCTC；ACCCACTCAACGGACTCTTT
qRT-JAZ5-F/R		AGAAGACGCCATCAACAAGG；CAGAGGGAAACCCATTAGGA
qRT-RNS1-F/R		CTTCCAAGCAGCCCTCAGTT；CCCAGTAGTATCCACGCACA
qRT-EXL2-F/R		GAACAACCTTTGGTGCTGAA；CCACGGTACATCTCAGTTGTCTTC
qRT-ASN1-F/R		TGGAGTGGGCTCTATCAGTG；AACGCTTCCTTAGTTCTTCATG
